# Olfactory Groove Meningioma Mimicking Late-Life Depression: A Case Report

**DOI:** 10.7759/cureus.113890

**Published:** 2026-08-03

**Authors:** André Silva, Catarina Matias-Martins, Inês S Fernandes

**Affiliations:** 1 Psychiatry and Mental Health, Unidade Local de Saúde do Médio Tejo, Tomar, PRT

**Keywords:** executive dysfunction, frontal lobe syndrome, late-life depression, meningioma, neuroimaging, olfactory groove

## Abstract

Late-life depression presenting with atypical features, progressive functional decline, behavioral changes, or poor response to treatment should prompt consideration of secondary causes. Olfactory groove meningiomas are slow-growing anterior skull base tumors that may remain clinically silent for prolonged periods and may present primarily with neuropsychiatric symptoms due to frontal lobe involvement.

We report the case of a 64-year-old woman with a progressive depressive presentation characterized by marked functional decline, apathy, abulia, behavioral disinhibition, impaired insight, and executive dysfunction. Symptoms were initially interpreted as a primary depressive disorder in the context of bereavement and were poorly responsive to psychopharmacological treatment. Neuroimaging revealed a large extra-axial anterior skull base mass centered on the olfactory groove, measuring approximately 63 mm in maximum diameter, with homogeneous contrast enhancement, marked mass effect on the frontal lobes, and extensive vasogenic edema. The patient underwent surgical resection in January 2024. Histopathological examination confirmed a World Health Organization grade 2 olfactory groove meningioma, with brain invasion and spontaneous necrosis. Postoperative imaging showed a small probable residual lesion, and the patient subsequently underwent adjuvant radiotherapy with 60 Gy in 30 fractions using volumetric modulated arc therapy. Follow-up imaging demonstrated a slight reduction in the residual lesion. Clinically, the patient showed postoperative improvement, with preserved orientation and language functions, no focal neurological deficits, and a return to functional independence in daily life, although residual anosmia and non-specific orthostatic symptoms persisted.

This case illustrates a strong clinical association between a large olfactory groove meningioma and a late-life neuropsychiatric presentation initially interpreted as depression. Such tumors should be considered when apparent late-life depression is accompanied by progressive functional deterioration, behavioral disinhibition, impaired insight, executive dysfunction, or poor response to adequate psychiatric treatment. Neuroimaging should be considered in these presentations to identify potentially treatable structural brain lesions.

## Introduction

Late-life depression requires careful clinical assessment, particularly when accompanied by atypical features, progressive functional decline, cognitive or behavioral changes, or poor response to adequate psychopharmacological treatment. Late-life depression is a heterogeneous clinical syndrome in which affective symptoms may coexist with apathy, cognitive impairment, and executive dysfunction. Executive dysfunction and slowed processing speed are considered common cognitive features of late-life depression and have been linked to disruption of frontal-subcortical networks and structural abnormalities involving frontal and temporal regions as well as white matter integrity. Although major depressive disorder is common in older adults, secondary causes, including neurological conditions, must be considered, as they may mimic primary psychiatric illness and lead to diagnostic delay [1,2].

Intracranial tumors, particularly those involving frontal brain regions, may present predominantly with psychiatric or cognitive symptoms. Frontal meningiomas may remain clinically silent until they reach substantial size, initially manifesting as progressive changes in personality, cognition, mood, motivation, or behavior rather than focal neurological deficits [3-5]. Although meningiomas are the most common primary intracranial tumors, olfactory groove meningiomas represent a relatively uncommon subgroup. Arising from the anterior skull base, they typically grow slowly and may reach large dimensions before diagnosis because of their insidious progression and initially subtle clinical manifestations [6].

Because of their proximity to anterior frontal structures and related networks, olfactory groove meningiomas may present with apathy, abulia, disinhibition, impaired insight, executive dysfunction, cognitive decline, depression, psychotic-like symptoms, anosmia, headache, visual symptoms, or seizures [6-10]. These psychiatric manifestations may precede localizing neurological signs, leading to their misattribution to a primary psychiatric disorder, particularly when the initial neurological examination is unremarkable.

We report the case of a 64-year-old woman with an apparent late-life depressive presentation accompanied by behavioral change, functional decline, and executive dysfunction. She was initially managed as having a primary psychiatric disorder but was later found to have a large olfactory groove meningioma. This case highlights the importance of considering structural brain lesions in older adults presenting with apparent late-life depression, particularly when depressive symptoms are accompanied by progressive behavioral change, executive dysfunction, impaired insight, or other features suggestive of a frontal lobe syndrome.

## Case presentation

A 64-year-old woman was brought to the psychiatric emergency department by her son because of progressive depressive symptoms and marked functional decline. The clinical picture had evolved over approximately four years and was characterized by apathy, anhedonia, abulia, reduced initiative, sleep and appetite disturbances, and significant impairment in occupational and domestic functioning. Shortly before presentation, the patient had discontinued psychopharmacological treatment because of a perceived lack of benefit, despite previous trials of antidepressant and adjunctive medications at adequate doses. Her psychiatric history included a depressive episode approximately 20 years earlier, temporally related to occupational stress, which had fully remitted with pharmacological treatment. She had no known history of neurological disease or other relevant medical comorbidities.

The current depressive presentation began following the death of her partner and was initially interpreted as a grief-related depressive disorder. Over the year preceding her emergency presentation, the patient developed progressive behavioral changes, including marked clinophilia and loss of occupational functioning. At home, she spent most of the day in bed using her mobile phone or watching online videos, neglected household tasks, and was unable to prepare meals, relying predominantly on readily available high-calorie foods. Collateral history revealed increasing behavioral disinhibition, inappropriate social comments, and the fabrication of implausible narratives. When confronted, the patient provided poorly systematized explanations with delusional content, attributing her inability to function to the influence of “spirits.” She demonstrated limited insight into her condition and exhibited affective incongruence, with episodes of inappropriate laughter during the clinical interview.

A bedside cognitive examination performed by the psychiatry team in the emergency setting revealed easy distractibility, together with predominantly executive and visuoconstructive dysfunction. The patient was unable to complete the clock-drawing test correctly, failed to copy intersecting pentagons, and demonstrated concrete thinking with impaired abstraction on proverb interpretation and similarity tasks. A formal quantitative cognitive assessment using a validated instrument, such as the Mini-Mental State Examination or the Montreal Cognitive Assessment, was not performed at presentation; therefore, no baseline quantitative cognitive score was available.

**Figure 1:**
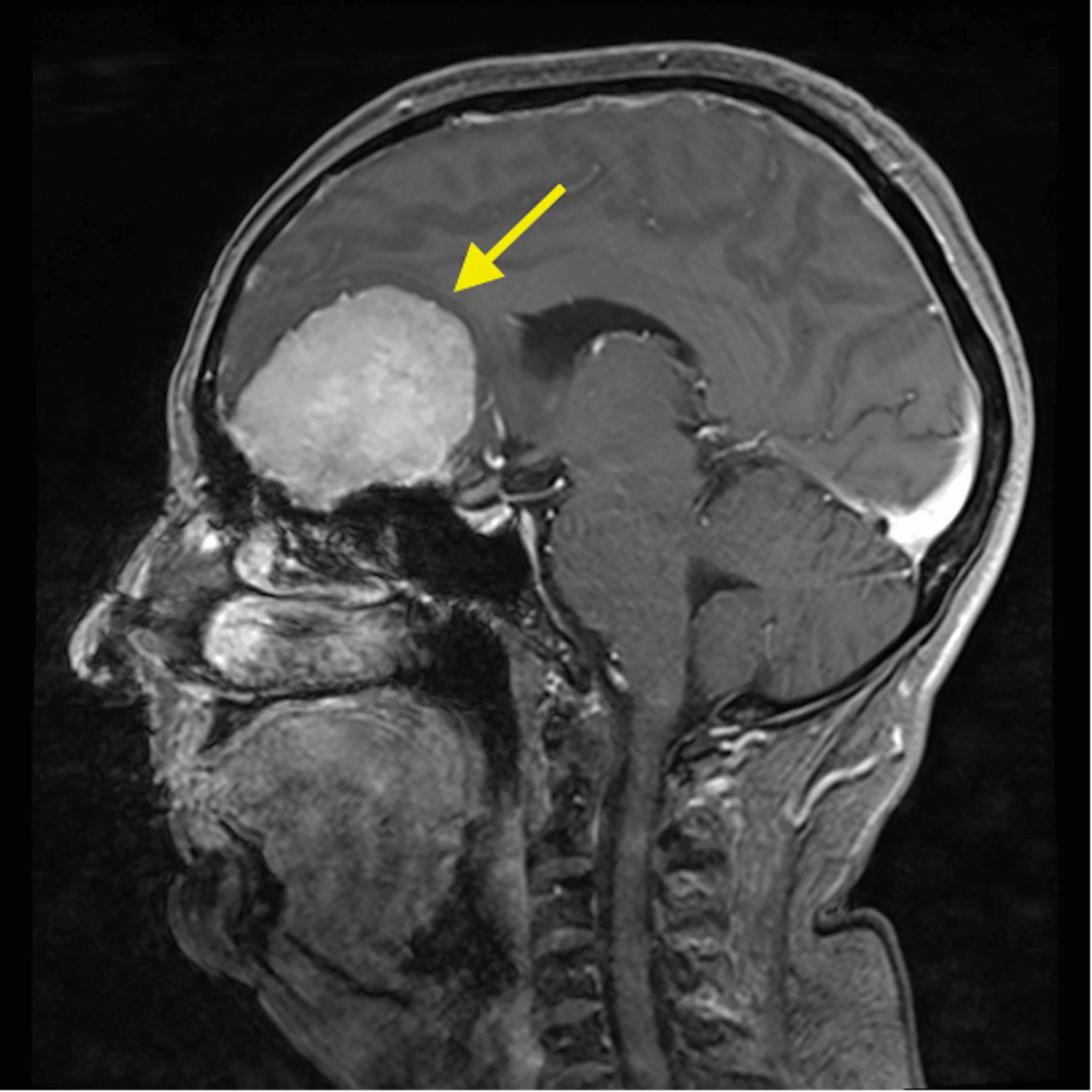
Preoperative post-contrast sagittal T1-weighted brain MRI A large, well-circumscribed, lobulated extra-axial mass centered on the olfactory groove demonstrates avid, predominantly homogeneous enhancement. The lesion causes marked mass effect on the frontal lobes and compression of the adjacent ventricular system. The solid arrow indicates the tumor MRI: magnetic resonance imaging

**Figure 2:**
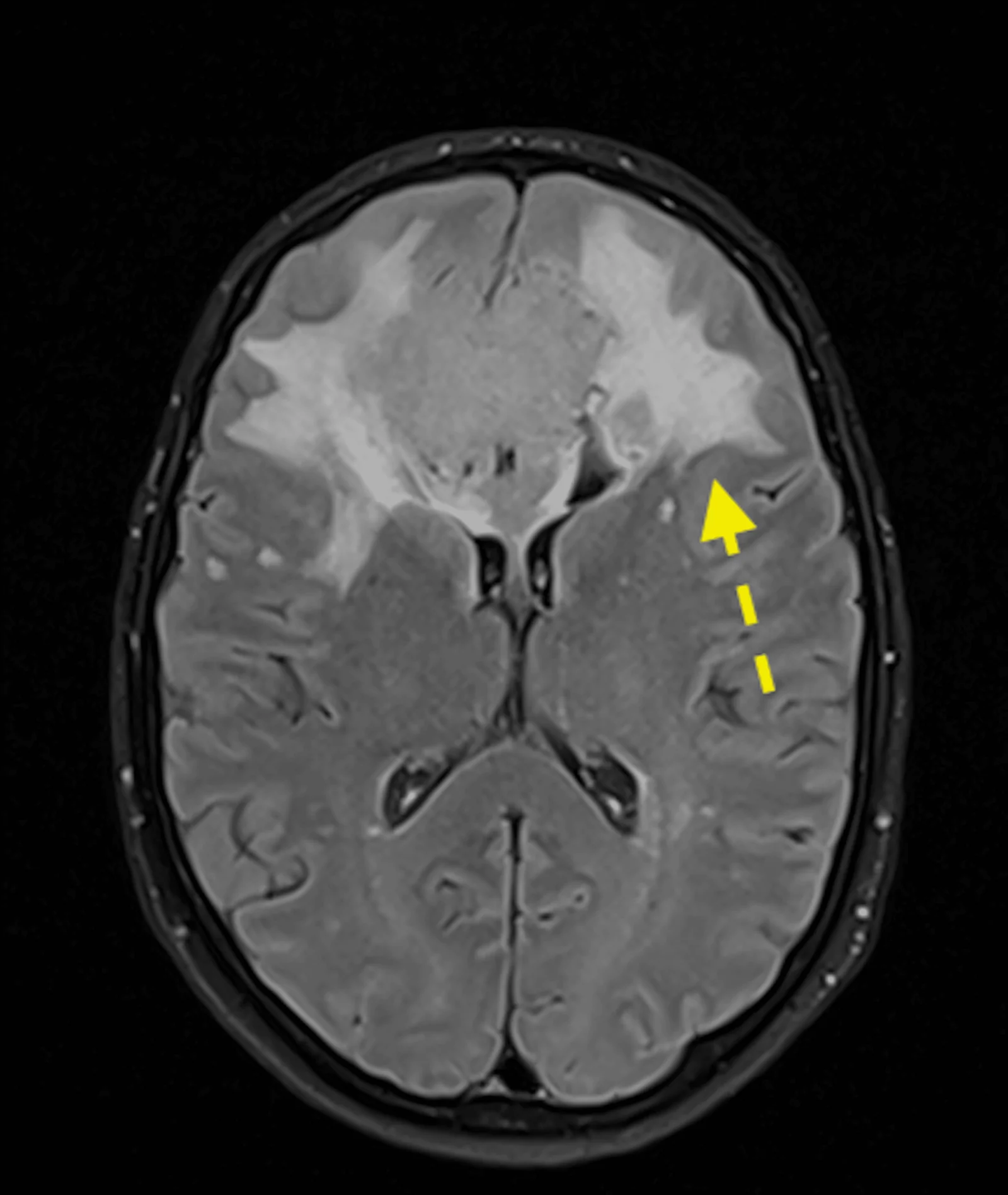
Preoperative axial T2-weighted FLAIR brain MRI Extensive bilateral frontal vasogenic edema, greater on the left, is seen adjacent to the anterior cranial fossa mass, with associated sulcal effacement and local mass effect. The dashed arrow indicates the peritumoral edema FLAIR: fluid-attenuated inversion recovery; MRI: magnetic resonance imaging

Given the atypical presentation, late-life onset, poor treatment response, and prominent behavioral and executive dysfunction, neuroimaging was pursued. Brain MRI revealed a large extra-axial mass centered on the olfactory groove and anterior skull base, measuring approximately 57 × 63 × 47 mm, with a maximum diameter of 63 mm. On post-contrast sagittal T1-weighted imaging, the lesion showed avid, predominantly homogeneous enhancement and produced marked frontal mass effect with compression of the adjacent ventricular system (Figure 1). Axial T2-weighted FLAIR imaging demonstrated extensive bilateral frontal vasogenic edema, greater on the left, with associated sulcal effacement and local mass effect (Figure 2). These findings provided an anatomically plausible structural correlate for the patient’s behavioral, motivational, and executive dysfunction.

**Figure 3:**
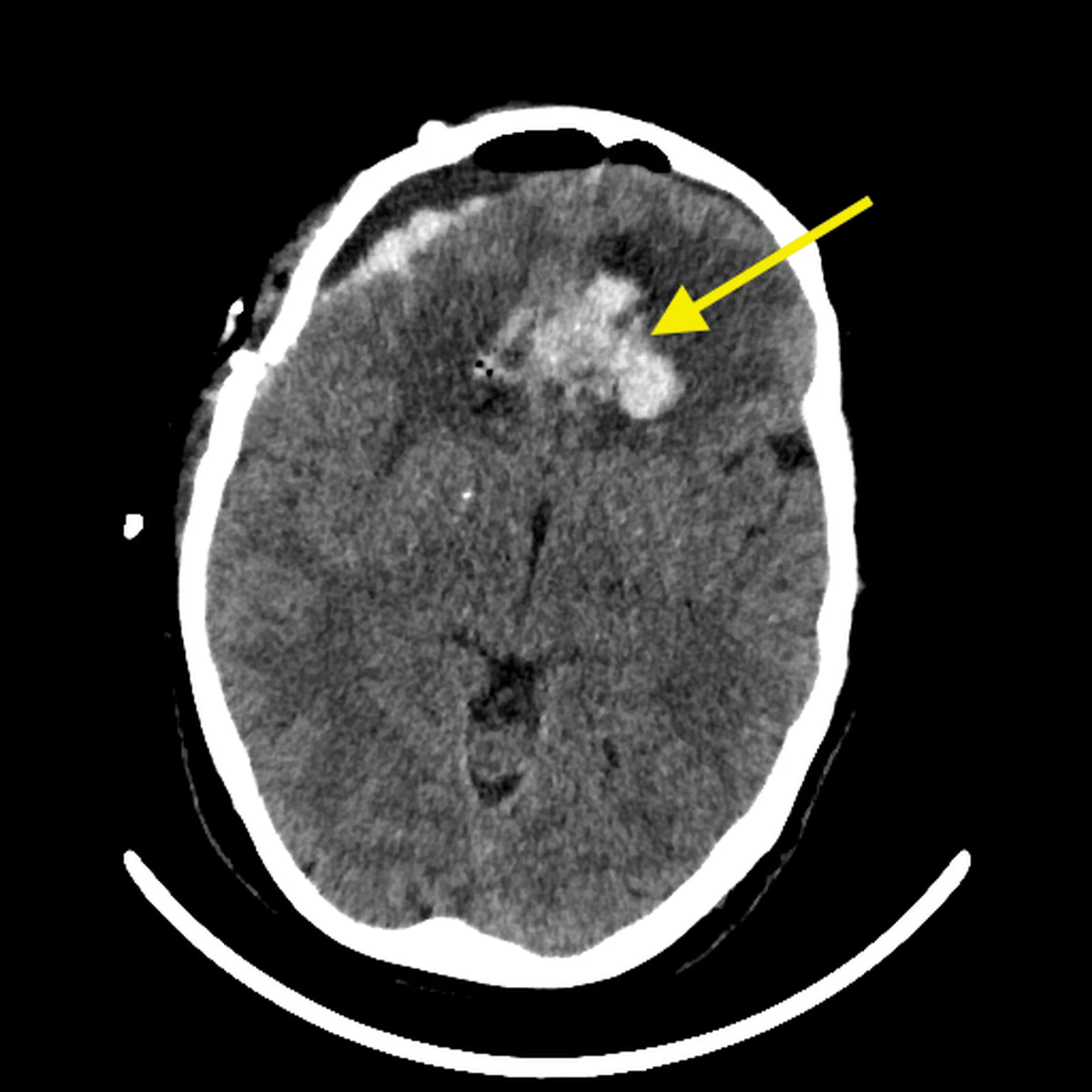
Postoperative non-contrast CT obtained on day 1 Axial CT image demonstrating a hematoma within the surgical cavity following right fronto-orbital craniotomy, without significant mass effect or relevant midline shift. The solid arrow indicates the postoperative hematoma CT: computed tomography

The patient was admitted to the neurosurgery department and underwent tumor resection through a right fronto-orbital craniotomy on day 1. Postoperative computed tomography showed a hematoma within the surgical cavity, without significant mass effect or relevant midline shift (Figure 3). Histopathological examination confirmed a World Health Organization grade 2 olfactory groove meningioma. The tumor showed brain invasion and small foci of spontaneous necrosis, without relevant mitotic activity or other features of anaplasia. Molecular analysis did not detect a TERT promoter mutation, and fluorescence in situ hybridization did not demonstrate homozygous deletion of CDKN2A. The Ki-67 proliferation index was not reported in the available histopathological documentation.

**Figure 4:**
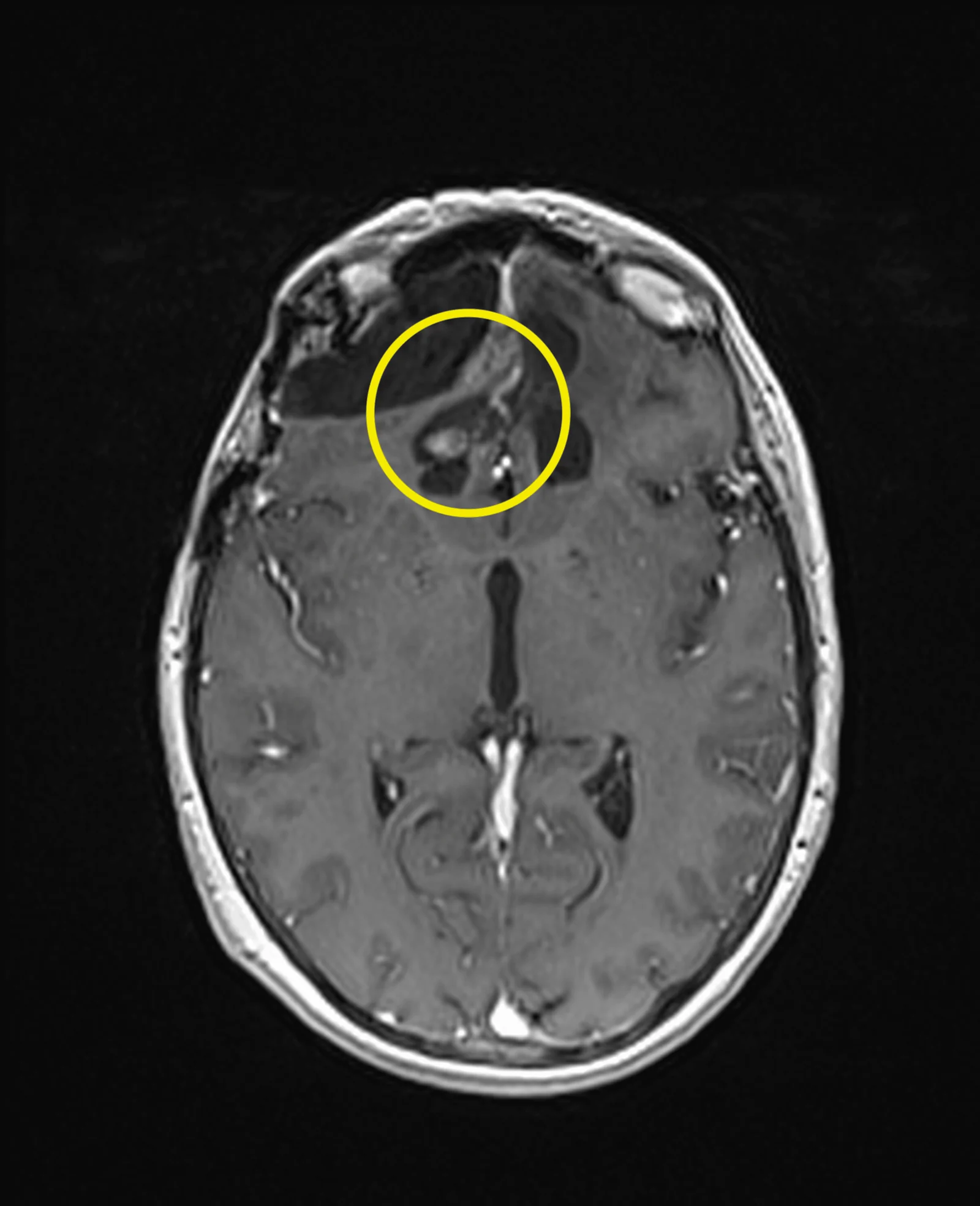
Postoperative post-contrast T1-weighted brain MRI obtained on day 136 Axial post-contrast T1-weighted MRI demonstrating postoperative changes after right fronto-orbital craniotomy. The yellow circle highlights a small left anteroinferior parafalcine residual enhancing lesion, without associated mass effect MRI: magnetic resonance imaging

Follow-up brain MRI, performed on postoperative day 136, showed postoperative changes after a right fronto-orbital craniotomy, including an extra-axial frontobasal collection without significant midline shift, persistent left anterior frontal T2/FLAIR hyperintensity, likely representing gliotic change secondary to previous vasogenic edema, and a small left anteroinferior parafalcine nodular enhancing lesion, interpreted as a probable residual tumor, without associated mass effect (Figure 4).

**Figure 5:**
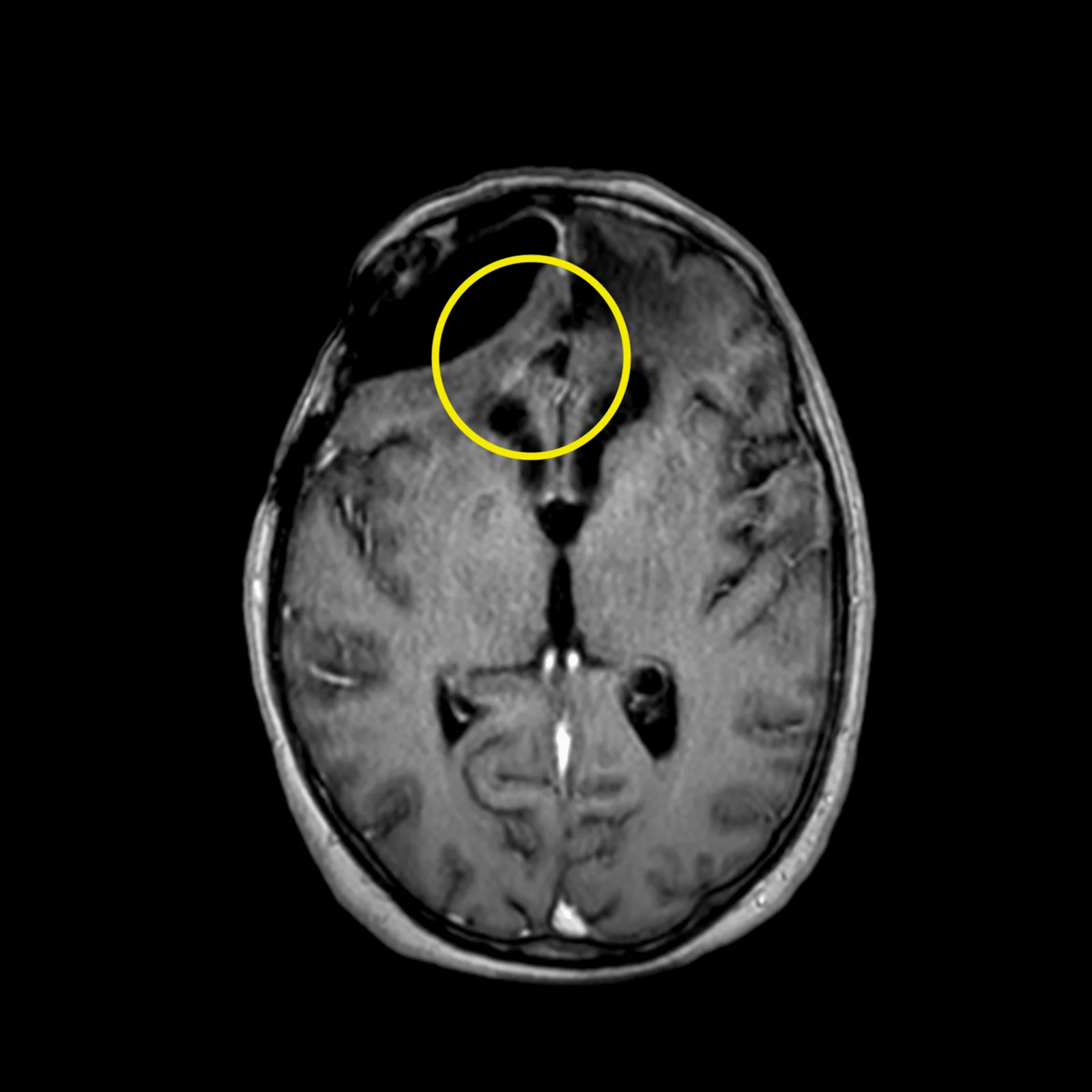
Follow-up post-contrast T1-weighted brain MRI obtained on day 607 Axial post-contrast T1-weighted MRI demonstrating slight interval reduction and stability of the residual left frontobasal enhancing lesion, highlighted by the yellow circle, following adjuvant radiotherapy MRI: magnetic resonance imaging

The patient was referred for adjuvant radiotherapy and received a total dose of 60 Gy in 30 fractions using volumetric modulated arc therapy between approximately postoperative day 160 and postoperative day 220. She subsequently remained under clinical and imaging surveillance. Follow-up MRI, performed on postoperative day 607, showed a slight interval decrease in the size of the residual left frontobasal meningioma, measuring 13 × 9 mm compared with 15 × 10 mm on the previous examination, with otherwise stable postoperative findings (Figure 5).

Following surgery, the patient showed marked neuropsychiatric improvement. Apathy and abulia progressively diminished, delusional ideas disappeared, behavioral disinhibition resolved, and she regained autonomy in activities of daily living. At neurological follow-up, she was awake, calm, cooperative, and oriented, with fluent and appropriate speech. Naming, comprehension, and repetition were preserved. There were no visual field defects on confrontation testing, cranial nerve deficits, motor weakness, sensory deficits, cerebellar signs, or gait abnormalities.

At subsequent follow-up, the patient remained clinically stable, lived independently at home, and continued under clinical and imaging surveillance. Residual anosmia persisted, although she occasionally reported brief, transient olfactory sensations. She also described intermittent orthostatic dizziness and blood pressure fluctuations following radiotherapy. No focal neurological deficits or recurrence of psychotic or disinhibited behavior was documented. At the most recent follow-up, her regular medication included simvastatin and oral iron supplementation. Levetiracetam, which had been administered during the postoperative period, had been discontinued.

## Discussion

This case illustrates how an olfactory groove meningioma may present predominantly with depressive, behavioral, and dysexecutive symptoms, leading to prolonged misattribution to a primary psychiatric disorder. The insidious progression, initial temporal association with bereavement, previous history of depression, and absence of early focal neurological signs contributed to diagnostic delay, highlighting the clinical challenge of distinguishing primary psychiatric illness from neuropsychiatric manifestations of structural brain disease. In older adults, stressful life events such as the loss of a partner may constitute a diagnostic trap, as clinicians, relatives, and caregivers may attribute emerging affective, cognitive, and behavioral symptoms to grief or psychosocial distress, potentially overlooking organic red flags. In the present case, the progressive functional decline, apathy, abulia, behavioral disinhibition, impaired insight, psychotic-like explanations, and objective executive dysfunction were atypical for an uncomplicated depressive or bereavement-related syndrome and ultimately prompted neuroimaging.

Similar diagnostic challenges are well documented in patients with olfactory groove and other frontal meningiomas presenting with psychiatric symptoms. These tumors may produce prolonged cognitive, affective, and behavioral manifestations before focal neurological deficits emerge, including depression, apathy, psychosis, personality change, disinhibition, impaired insight, attention and memory deficits, and executive dysfunction [4,7,9-14]. Symptoms may initially be attributed to depression, stress, aging, personality change, psychosis, or dementia. A recent systematic review found that cognitive, affective, and behavioral symptoms are frequent in patients with olfactory groove meningiomas, although their reported prevalence varies widely across studies [6].

Several case reports further illustrate the risk of initial misdiagnosis as a primary psychiatric disorder. For example, Mumoli et al. reported a patient initially diagnosed with bipolar disorder who was later found to have a giant frontal meningioma, with improvement of psychiatric symptoms after resection [5]. Suradom et al. similarly described schizophrenia-like symptoms associated with a frontal meningioma, which also improved following surgical removal [14]. These cases illustrate the range of neuropsychiatric presentations reported in frontal meningiomas. Such presentations should prompt consideration of a structural frontal lobe syndrome, particularly when symptoms emerge later in life, progressively worsen, or do not follow the expected course of a primary psychiatric disorder.

The patient’s presentation overlapped with dysexecutive phenotypes described in late-life depression, including apathy, disability, reduced insight, and impaired executive functioning [1,2]. However, the progressive course, prominent behavioral disinhibition, psychotic-like explanations, poor treatment response, and structural lesion on neuroimaging strongly supported the interpretation of a secondary neuropsychiatric syndrome. Structural MRI studies in late-life depression have linked cognitive impairment to frontal and temporal abnormalities and reduced white matter integrity [15]. In the present case, the large anterior skull base mass, bilateral frontal mass effect, and extensive vasogenic edema provided a plausible and anatomically coherent structural mechanism for disruption of frontal networks.

The symptom constellation was consistent with a frontal lobe syndrome involving multiple prefrontal circuits. Apathy, abulia, reduced initiative, and diminished goal-directed behavior predominantly suggest disruption of the anterior cingulate and medial prefrontal circuits. Behavioral disinhibition, affective incongruence, personality change, and impaired insight are more consistent with orbitofrontal/ventromedial prefrontal dysfunction, whereas executive impairment points to dorsolateral prefrontal circuit disruption. The coexistence of these features is compatible with the bilateral frontal compression and edema demonstrated on neuroimaging. The distinction between depression and apathy is clinically relevant. Although both may involve reduced motivation, withdrawal, and diminished activity, prominent loss of initiative, behavioral inertia, impaired insight, and executive dysfunction should raise suspicion for frontal network dysfunction [16]. In this patient, the combination of apathy and abulia with impaired abstraction, concrete thinking, inappropriate laughter, and social disinhibition suggested a broader frontal lobe syndrome rather than a purely affective disorder.

Although olfactory groove meningiomas are generally slow-growing tumors, the patient’s more pronounced clinical deterioration during the final year may have reflected not only increasing mass effect but also the biological and histopathological characteristics of the lesion. Histological examination demonstrated a World Health Organization grade 2 meningioma with brain invasion. Brain invasion, together with extensive vasogenic edema and bilateral frontal compression, may have contributed to a more rapid disruption of frontosubcortical networks and the subsequent worsening of cognition, behavior, and functional independence. This apparent acceleration is therefore not necessarily inconsistent with the otherwise indolent natural history commonly associated with olfactory groove meningiomas.

Several red flags should therefore be emphasized: late-life onset or marked change in psychiatric symptoms, progressive functional deterioration, poor response to adequate treatment, behavioral disinhibition, impaired insight, psychotic-like or poorly systematized explanations, objective executive dysfunction, and olfactory symptoms. Anosmia may be an early but underrecognized manifestation of olfactory groove meningioma and should be actively assessed when anterior skull base pathology is suspected [6]. The presence of such features should lower the threshold for neuroimaging, even in patients with a previous psychiatric history or when symptoms initially appear temporally related to bereavement or another stressful life event.

The World Health Organization grade 2 diagnosis warranted ongoing neurosurgical and oncological follow-up because of the risk of recurrence, particularly in the presence of residual disease and brain invasion. Adjuvant radiotherapy may improve progression-free survival in this context [17]. In this case, postoperative MRI suggested a small residual lesion, and the patient underwent radiotherapy with 60 Gy in 30 fractions. Subsequent MRI showed a slight reduction in the residual lesion, and she remains under clinical and imaging surveillance.

This case report has several limitations. Formal quantitative cognitive assessment using a validated instrument, such as the Mini-Mental State Examination or Montreal Cognitive Assessment, was not performed before or after surgery, precluding objective longitudinal comparison of cognitive performance. The Ki-67 proliferation index was also not reported in the available histopathological documentation. The neuropsychiatric outcome was therefore assessed primarily through clinical observation, collateral information, functional recovery, and longitudinal follow-up. Furthermore, causal inference is inherently limited in a single case report.

Although the anatomical localization of the tumor, the associated bilateral frontal mass effect and vasogenic edema, the clinical features of frontal network dysfunction, and the marked postoperative improvement strongly support the meningioma as the principal explanation for the neuropsychiatric presentation, a definitive causal relationship cannot be established. A coexisting primary late-life depressive disorder or additional psychiatric and psychosocial contributing factors, including bereavement, cannot be completely excluded. Nevertheless, the improvement in apathy, abulia, behavioral disinhibition, delusional ideas, and functional independence following tumor treatment supports a strong clinical association between lesion treatment and neuropsychiatric recovery.

This case reinforces the importance of maintaining a broad differential diagnosis in patients presenting with late-life depression. Atypical features, progressive dysexecutive decline, behavioral change, impaired insight, and treatment resistance should prompt investigation for secondary causes. Early neuroimaging may identify potentially treatable structural lesions and reduce the risk of prolonged diagnostic delay.

## Conclusions

This case report highlights how an olfactory groove meningioma may initially mimic late-life depression while progressively producing a broader frontal neuropsychiatric syndrome characterized by apathy, abulia, behavioral disinhibition, impaired insight, executive dysfunction, and functional decline. The clinical course, anatomical findings, and postoperative improvement suggest that the meningioma played a major role in the patient’s presentation, although other psychiatric and psychosocial factors may also have contributed to the overall clinical picture. Late-onset depressive symptoms, progressive functional deterioration, poor response to treatment, and the emergence of behavioral or executive dysfunction should prompt consideration of secondary neurological causes. Neuroimaging plays a crucial role in identifying potentially treatable structural lesions that may masquerade as primary psychiatric disorders.
